# Effect of self-activating phthalocyanine gel on palatal donor site healing, inflammatory biomarkers, somatosensory and patient-centered outcomes: a randomized split-mouth clinical trial

**DOI:** 10.1007/s00784-026-07117-x

**Published:** 2026-09-12

**Authors:** Caique Andrade Santos, Rafael Sponchiado Cavallieri, Maria Carolina Sementille, Thiago José Dionísio, Rafaela Stocker Salbego, Carla Andreotti Damante, Leonardo Rigoldi Bonjardim, Mariana Schutzer Ragghianti Zangrando

**Affiliations:** 1 Bauru School of Dentistry, Department of Prosthodontics and Periodontics, University of São Paulo, Vila Nova Cidade Universitária, Al. Dr. Octávio Pinheiro Brisolla, 9-75, Bauru, São Paulo, 17012-901 Brazil; 2 Bauru School of Dentistry, Departament of Biological Sciences, ZIP CODE, University of São Paulo, Vila Nova Cidade Universitária, Al. Dr. Octávio Pinheiro Brisolla, 9-75, Bauru, São Paulo, 17012-901 Brazil

**Keywords:** Subepithelial connective tissue graft, Palatal donor site, Wound healing, Phthalocyanine derivative, Randomized clinical trial, Quantitative sensory testing

## Abstract

**Objectives:**

To evaluate effects of self-activating phthalocyanine (PHY) gel on palatal donor site after subepithelial connective tissue graft (SCTG) harvesting, considering clinical outcomes, inflammatory biomarkers, patient-centered outcomes, and quantitative somatosensory changes.

**Materials and methods:**

Twenty participants were included in this randomized, crossover, split-mouth clinical trial. Donor sites received topical PHY gel (PHY group-PHYG) and placebo gel (control group-CG). Clinical parameters (residual wound area-RWA, wound epithelialization-WE, residual remodeling area-RRA and tissue thickness-TM) were assessed at baseline, 7, 14, 30, and 60 days. Patient-centered outcomes were recorded during 14 postoperative days and inflammatory biomarkers at 3 and 7 days postoperatively. Mechanical detection threshold (MDT) and mechanical pain threshold (MPT) were evaluated at baseline, 2 months, and 6 months.

**Results:**

PHYG and CG exhibited similar clinical outcomes, but PHYG demonstrated superior performance in reducing RRA. Both groups presented positive postoperative progression of patient-centered outcomes. IL-10 levels showed a significant increase in PHYG, suggesting a more favorable anti-inflammatory response. MDT remained stable but, donor sites exhibited transient hypersensitivity (MPT) at 2 months.

**Conclusions:**

PHYG and CG protocols promoted satisfactory healing of palatal donor sites. Most clinical parameters and patient-centered outcomes were similar between groups. The PHYG presented reduced RRA and increased IL-10 levels. Donor sites exhibited transient hypersensitivity, which tended to resolve over time.

**Clinical Relevance:**

Both postoperative protocols resulted in favorable healing of palatal donor sites following SCTG harvesting, with comparable clinical and patient-centered outcomes. Quantitative sensory testing indicates that hypersensitivity may occur during healing, but sensory function tends to recover progressively.

## Introduction

Subepithelial connective tissue grafts (SCTGs) are indicated for different procedures involving teeth, implants, and edentulous ridges [1]. Several techniques have been described for SCTG harvesting, including the parallel incision, single-incision, and de-epithelialized free gingival graft techniques [2–4]. Despite their clinical effectiveness, palatal donor sites are associated with relevant postoperative morbidity, including pain, delayed healing, bleeding, and potential sensory disturbances, which may negatively impact quality of life. Aiming to improve postoperative period, particularly considering graft donor areas, several methods for protecting the palate have been investigated. These strategies include purely physical barriers, biologically active agents, as well as combinations of both approaches. Among these, the use of surgical dressing [5], modified Hawley plate [6], collagen sponge with cyanoacrylate [7], photobiostimulation [8], fibrin-rich plasma [9], hyaluronic acid and herbal extracts [10]. Despite this diversity, no consensus has been reached regarding a gold-standard protocol for palatal donor site management. These strategies can present limitations, such as lack of bioactive properties, persistent patient discomfort, or limited ability to modulate the biological healing process.

Then, the lack of consensus regarding the ideal method for palatal protection encourages the search for new alternatives. These approaches should not only provide mechanical protection, but also exhibit bioactive properties capable of enhancing the reconstructive process of the palatal wound [11, 12]. Biological effects of phthalocyanines derives from studies using photodynamic therapy (PDT) [13, 14]. Upon light activation, phthalocyanines generate reactive oxygen species (ROS), which play a key role in cellular signaling, antimicrobial activity, angiogenesis, and tissue remodeling during wound healing. Self-activating phthalocyanine (PHY) derivatives is applied without light activation but also promote the controlled generation of ROS. This compound may also influence the local inflammatory response and tissue healing. In vitro studies have shown that PHY has antibacterial, antifungal, antibiofilm, antiviral, low toxicity properties and not negatively interfere in wound healing [15–18]. Clinical studies have shown positive effects on periodontal treatment and ulcer healing [19–21]. Therefore, these properties suggest a potential role of PHY in enhancing oral wound healing beyond conventional mechanical protection.

Additionally, assessment of patient-centered outcomes is extremely relevant, particularly regarding their sensory perceptions. In this context, only five studies evaluated sensory changes in palatal donor sites using different methods. Sensibility assessments [22, 23] demonstrated no loss of sensation, temporary paresthesia or paresthesia in SCTG donor area. Another study reported a persistent numbness or a rough palatal surface postoperatively [24]. Qualitative assessment of the somatosensory profile demonstrated variations of hyposensitivity and hypersensitive in palatal donor sites [25, 26]. Accordingly, given the heterogeneity in methodologies and outcomes, further studies employing quantitative evaluations of these potential sensorial changes are necessary.

To the best of our knowledge, no previous randomized clinical trial has simultaneously investigated clinical outcomes, inflammatory biomarkers, patient-centered outcomes, and quantitative somatosensory changes in palatal donor sites. Therefore, the aim of this study was to investigate the effects of PHY gel on palatal donor site healing following SCTG harvesting through an integrated assessment of these parameters. The null hypothesis was that PHY gel would not result in differences in the percentage of residual wound area (RWA) of palatal donor sites when compared with control. The alternative hypothesis was that PHY gel would result in superior improve of this outcome during palatal wound healing. The findings of this study may contribute to optimizing postoperative management strategies and improving patient care in periodontal plastic surgery.

## Material & methods

This superiority randomized, controlled, crossover, blinded, split-mouth clinical trial was conducted at the Periodontics clinic of Bauru School of Dentistry, University of São Paulo (FOB-USP) according to CONSORT guidelines. The study was approved by the Research Ethics Committee for Human Studies at FOB-USP (CAAE: 19692019.1.0000.5417) and registered in the Brazilian Registry of Clinical Trials (ReBEC – RBR-93ccq38). All participants provided written informed consent prior to enrollment.

Patients with clinically healthy palatal tissue who required bilateral SCTG for root coverage procedures were included. The exclusion criteria were use of palatal coverage prostheses, previous history of palatal graft harvesting, smoking, pregnancy or lactation, diabetes mellitus and use of medications that could interfere with the healing process (such as anticonvulsants, antihypertensives, hormonal contraceptives, or immunosuppressants). Patients in use of systemic antibiotic therapy or other antimicrobial mouthwashes during the study period, and poor oral hygiene (plaque index and bleeding index > 20%) were also excluded.

Surgical procedures were performed under optical magnification (3.5×) by the same clinician (RSC). Bilateral multiple gingival recessions involving 2 or 3 adjacent teeth were treated using the coronally advanced flap (CAF) associated with SCTG, harvested with de-epithelialization technique [27, 28]. Patients were standardized to present similar grafting demands on both the test and control sites. Grafts were harvested from the palatal premolar/molar region and standardized to 5 mm in width, a length corresponding to the mesiodistal distance of two or three adjacent teeth, and a thickness of approximately 1 mm. Due to the split-mouth design, bilateral grafting requirements were comparable within each patient, allowing intrapatient standardization of the surgical extension. In the palatal donor area, X-shaped sutures were performed using blue high-tenacity nonabsorbable monofilament polyamide sutures (Nylon Blue, Techsuture, Brazil).

The pharmacological protocol followed the guidelines established by the Periodontics Department of FOB-USP (Bauru School of Dentistry, University of Sao Paulo), consisting of a preoperative dose of 8 mg dexamethasone and 100 mg nimesulide twice daily (4 days). In case of pain, 750 mg paracetamol was recommended as rescue medication. Participants were not informed about which postoperative protocol was applied to each donor site and received written instructions regarding postoperative home care for the first 14 days. Two postoperative protocols were implemented according to group allocation:

- PHYG (Test group): Topical application of 0.1% PHY gel on the palatal wound and mouthrinse with iron phthalocyanine (0.12%);
- CG (Control group): Topical application of placebo gel (without PHY) on the palatal wound and mouthrinse alcohol-free chlorhexidine gluconate (0.12%).

Participants were allocated into 1:1 blocks to balance the order of protocol application (CG and PHYG) and reduce bias in subjective outcomes. Within each block, simple randomization was applied to determine the side (right or left) for the application of PHYG or CG. Allocation was concealed using opaque envelopes, which were opened only at the time of surgery. Randomization was performed using the Random Sequence Generator software (www.random.org) and Microsoft Excel. A washout period of 45 days was adopted, considering donor site evaluations (60 days postoperatively), although the literature recommends a minimum of 15 days [29–31].

**Fig. 1:**
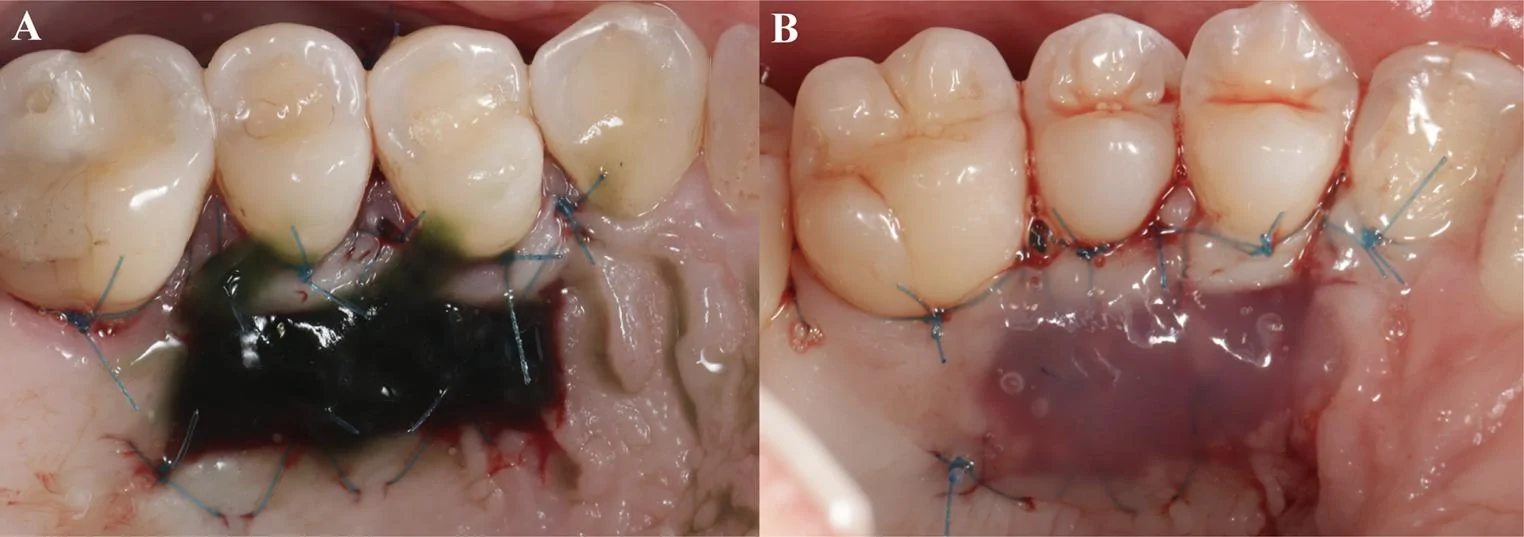
Application of gels to palatal donor sites following SCTG harvesting. (**A**) PHY gel in PHYG. (**B**) Placebo gel in CG

After surgical procedures, patients were given standardized instructions to floss and brush their teeth with provided fluoridated toothpaste, three times daily in the non-operated areas. Agents were applied in a standardized sequence as follows (Fig. 1):

- PHYG- first, mouthrinse (Iron phthalocyanine 0.12%) followed by gel application on palatal donor area (Iron phthalocyanine 0.1%);
- CG– first, mouthrinse (alcohol-free chlorhexidine gluconate 0.12%), followed by placebo gel application on palatal donor area. Chlorhexidine mouthrinse was used in CG because it is the most prescribed postoperative rinse following periodontal surgeries and for ethical reasons.

Mouthrinses were used twice daily (after lunch and dinner) for 60 s, 30 min after toothbrushing. Both gel formulations were manufactured to have similar consistency and were dispensed in identical, unlabeled tubes. Nevertheless, the presence of iron phthalocyanine may have resulted in slight differences in color between the formulations. To minimize the potential risk of unblinding, participants were not informed about which formulation corresponded to the placebo or the active compound, and no information regarding the expected appearance of the gels was provided. Gel application was performed with the fingertip (in an amount approximately the size of a pea) directly over the operated palatal wound, three times daily (after breakfast, lunch, and dinner). Following gel application, patients were instructed to refrain from eating or drinking for at least one hour. Both the mouthrinses and topical gels were used for 14 postoperative days in both groups. Sutures were removed after 7 days; however, participants were instructed to continue the assigned postoperative protocol until completion of the 14-day period.

The blinded examiner responsible for data collection (CAS) was previously calibrated, repeating primary outcome percentage of residual wound area (RWA) measurements in a set of patients (10%) on two separate occasions prior to the study. RWA was measured by outlining the wound margins, considering the non-epithelialized wound area. Intra-examiner reliability was assessed using the intraclass correlation coefficient, demonstrating excellent agreement (ICC = 0.95).

The sample size calculation was based on primary outcome (RWA), considered a paired design and the detection of a minimum clinically relevant difference of 15% between groups, assuming an estimated standard deviation of 25%, a two-sided significance level of 5% (α = 0.05), and a statistical power of 80% (β = 0.20). Based on these parameters, a minimum of 18 participants was required. To compensate for a potential dropout rate of 20%, the final sample size was set at 20 individuals. The parameters used for the calculation were derived from previously published data on palatal wound healing after SCTG harvesting [32].

Previous studies [32–34] served as a conceptual and methodological basis for the measurement of clinical parameters. Thus, some adaptations were implemented to address the specific objectives of the present study. Residual remodeling area (RRA) were assessed and measured based on the visualization of the surgically incised margins undergoing the healing process. Specifically, areas were considered part of the RRA when, despite being epithelialized, they exhibited a distinct coloration, appearing more erythematous compared to the adjacent tissues, indicating ongoing remodeling.

The WE parameter was derived through a mathematical calculation in which the RWA at baseline was used as the reference, and the RWA (i.e., the non-epithelialized area) at each postoperative assessment was subtracted from the baseline RWA. WE was calculated according to the following formula: WE (%) = [(RWA baseline − RWA at postoperative assessment) / RWA baseline] × 100.

**Fig. 2:**
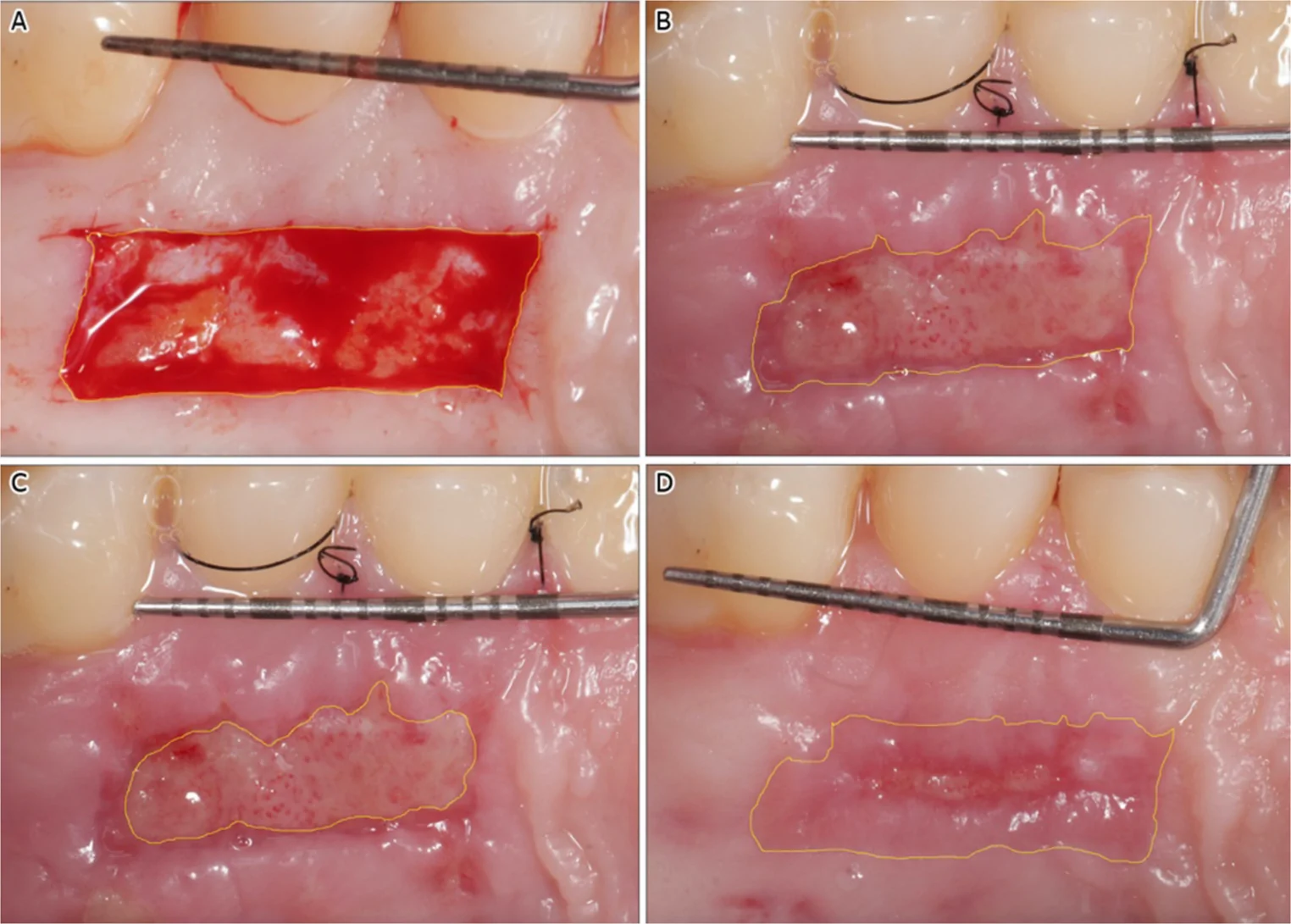
Clinical parameters of donor sites. (**A**) RWA after SCTG harvesting (baseline). (**B**) RRA at 7 days postoperatively. (**C**) Delimitation of RWA at 7 days. (D) Delimitation of RRA at 14 days postoperatively. The yellow outline indicates the area measured using ImageJ software

TM (tissue thickness) was measured on baseline and at 2 months post-surgery using a needle with an endodontic stopper and a digital caliper. RWA, WE and RRA were assessed at baseline, 7, 14, 30, and 60 days postoperatively using standardized photographs (constant light intensity, distance, and angle) and ImageJ software (NIH, Bethesda, USA) (Fig. 2).

All images were standardized prior to analysis using a calibration procedure based on a known reference distance (periodontal probe) to ensure consistent spatial scaling (mm). The region of interest (ROI) was defined for each image according to previously established anatomical landmarks, ensuring consistency across all samples. A standardized thresholding procedure was applied to distinguish the area of interest from the surrounding tissues. The same threshold range was used for all images to minimize operator-dependent variability. Segmentation was performed using semi-automated selection tools, followed by manual refinement when necessary to ensure accurate delineation of the measured area. All measurements were conducted by a single blinded calibrated examiner.

Patient-centered outcomes were assessed during the 14-day postoperative period using a diary [35] to record treatment perceptions and spontaneously report of any adverse effects. The diary included postoperative symptoms, pain and discomfort at the palatal donor sites, oral function (chewing difficulty), and interference with daily activities. Responses were obtained using visual analog scales (VAS). The number of analgesics consumed per day (rescue medication) was also recorded. Additionally, to ensure patient compliance, the diary contained a protocol adherence table, in which patients were instructed to mark “YES” or “NO” for each application performed, recording the number of brushings, rinses, and topical applications throughout the day. This monitoring allowed for objective verification of product use regularity and adherence to protocol.

Oral health-related quality of life was assessed using the validated 14-item short version of the Oral Health Impact Profile (OHIP-14), administered before surgical treatment and at 6 months of follow-up [36].

For the analysis of inflammatory biomarkers, exudative fluid was collected from the edges of the palatal wound using absorbent paper strips (Cellpack – AllPrime) at 3 and 7 days postoperatively. The donor area was dried, and eight absorbent paper strips were individually placed—two on each border of the surgical site—and left in place for 40 s. The strips were then stored in 2 mL Eppendorf tubes containing 1 mL of phosphate-buffered saline (PBS) with 0.01 g/mL SigmaFast™ Protease Inhibitor Tablet. All samples were stored at − 80 °C for subsequent lyophilization. According to the manufacturer’s instructions (MILLIPLEX^®^ Human Cytokine/Chemokine/Growth Factor Panel magnetic bead-based multiplex assay- HCYTA-60 K (Merck Millipore, Billerica, MA, USA), the lyophilized standards and controls were reconstituted in 250 µL of deionized water. For samples analyzed using the multiplex assay, the protocol recommended the use of assay buffer, and each well received 25 µL of the sample and 25 µL of assay buffer. Multiplex assay was used to determine the levels of IL-6 (interleukin-6), IL-10 (interleukin-10), PDGF-AA (platelet-derived growth factor AA), PDGF-AB/BB (platelet-derived growth factor AB/BB), TGF-α (transforming growth factor alpha), TNF-α (tumor necrosis factor alpha) and VEGF-A (vascular endothelial growth factor A), using a five-parameter polynomial equation via Xponent^®^ software (Millipore Corporation, Billerica, MA, USA). Total protein content of each sample was determined by the Bradford assay (B6916, Sigma-Aldrich^®^, St. Louis, Missouri, USA). Accordingly, the total amount of each biomarker was expressed in pg/mg [34, 37].

**Fig. 3:**
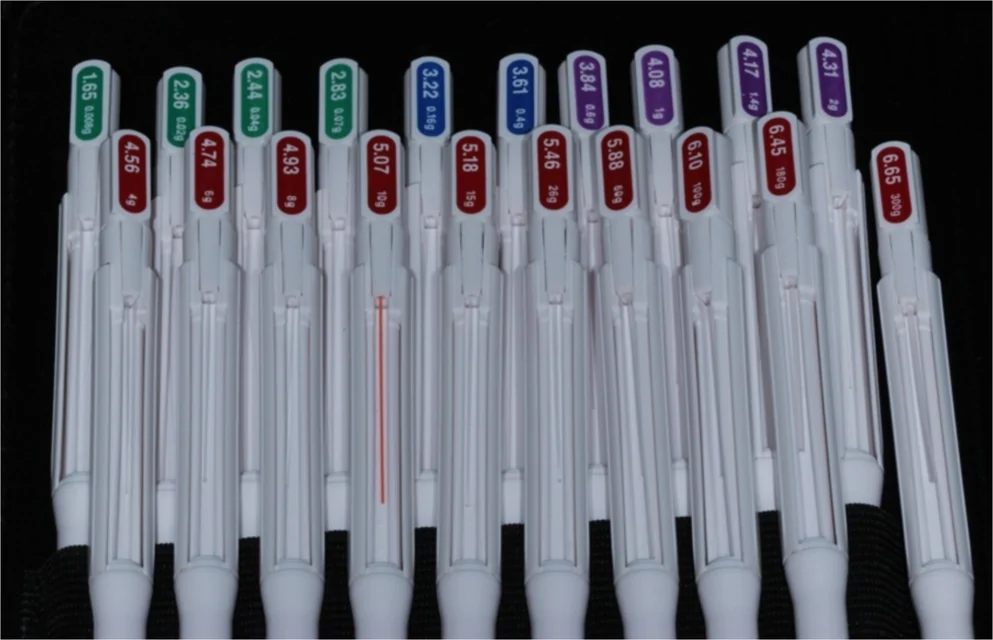
Von Frey filaments for somatosensory assessment of the graft recipient site

**Fig. 4:**
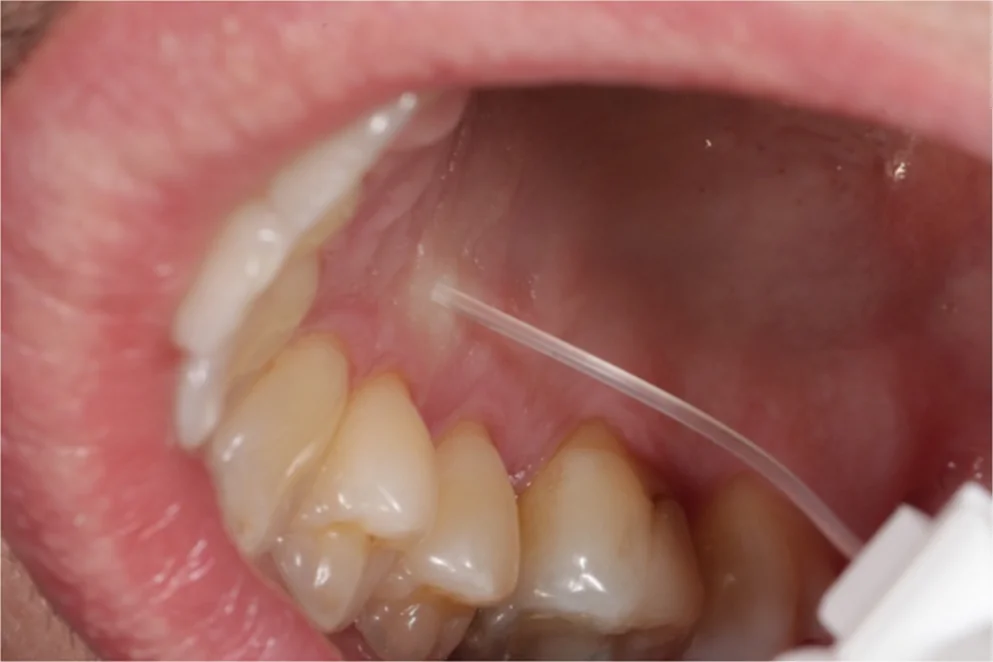
Quantitative sensory test using Von Frey filaments at palatal donor site for the evaluation of mechanical detection threshold (MDT) and mechanical pain threshold (MPT)

The quantitative evaluation of the somatosensory profile of the donor sites was performed using Mechanical Detection Threshold (MDT) and Mechanical Pain Threshold (MPT) tests, applied at baseline, 2 months, and 6 months after the surgical procedure. The MDT test employed Semmes-Weinstein (Von Frey) nylon monofilaments to determine participants’ tactile threshold [38] (Fig. 3). The force exerted by the filaments ranged from 0.008 g/mm² to 300 g/mm² (Fig. 4). Prior to testing, participants were instructed to keep their eyes closed and verbally indicate when they perceived a “light touch” on the assessed area. The contact area on the palate was defined as 1.5 to 2 mm from the gingival margin of the premolars, corresponding to the graft donor site. The test started with the thinnest filament (0.008 g/mm²), followed by sequential application of progressively thicker filaments until the participant verbally reported perception of the light touch (positive stimulus). From that point, the sequence was reversed, applying filaments of decreasing intensity until the participant no longer perceived the tactile stimulus (negative stimulus). This procedure was repeated until five negative (descending) and five positive (ascending) responses were obtained, and the geometric mean of the repetitions was calculated [39–41]. The MPT test followed the same methodology using the same adapted monofilaments but aimed to assess the mechanical pain threshold. Participants were instructed to verbally report when they perceived a sensation described as a “prickling, stabbing, or slightly painful prick”. Stimulus application and result calculation followed the same criteria as those used for MDT [39].

The statistical analysis considered a two-factor factorial design: the group factor, with two levels (control and test), and the time factor, treated as a repeated measure with levels varying according to the dependent variable assessed. Clinical and patient-related outcomes were primarily analyzed using two-way repeated measures analysis of variance (ANOVA-RM) in Statistica 10.0, considering the effects of group, time, and their interaction. Tukey’s post hoc test was applied when appropriate for multiple comparisons over time. Considering the split-mouth design, paired t-tests were additionally used for specific pairwise comparisons between control and test sites at individual evaluation periods when necessary.

The statistical analysis of biomarkers compared the concentrations of the analytes (IL-6, IL-10, PDGF-AA, PDGF-AB/BB, TNF-α, and VEGF-A) on postoperative days 3 and 7. The percentage change in the levels of biomarkers was compared between groups. For data that did not show a normal distribution, the nonparametric Wilcoxon test was applied. For variables with a normal distribution, the paired t-test was used, and the results were expressed as mean and standard deviation. The Pearson correlation test was employed to evaluate the associations between biomarker concentrations, clinical parameters, and patient-related outcomes. The quantitative sensory analysis was performed using Repeated-measures analysis of variance (ANOVA-RM). The significance level was set at *p* < 0.05.

## Results

**Fig. 5:**
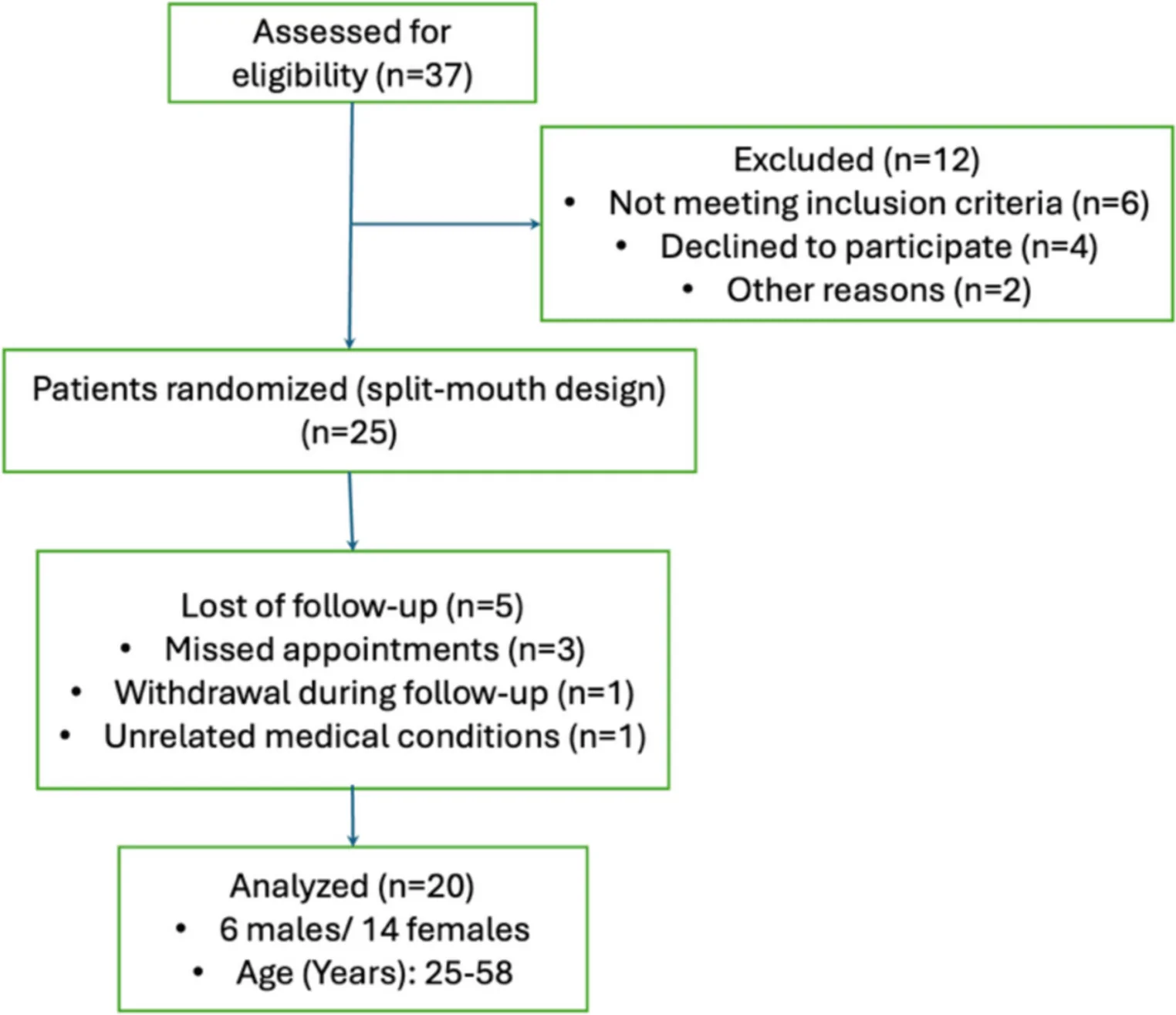
Flow diagram of participant selection, randomization and follow-up

Twenty-seven participants were initially enrolled. After withdrawals and exclusions during the follow-up period, 20 participants completed the study (Fig. 5). All participants presented periodontal health or periodontal stability prior to surgery, with plaque index and bleeding index < 20%. A total of 40 surgical procedures were performed, with 20 donor sites allocated to each group (CG and PHYG).

## Clinical outcomes

**Fig. 6:**
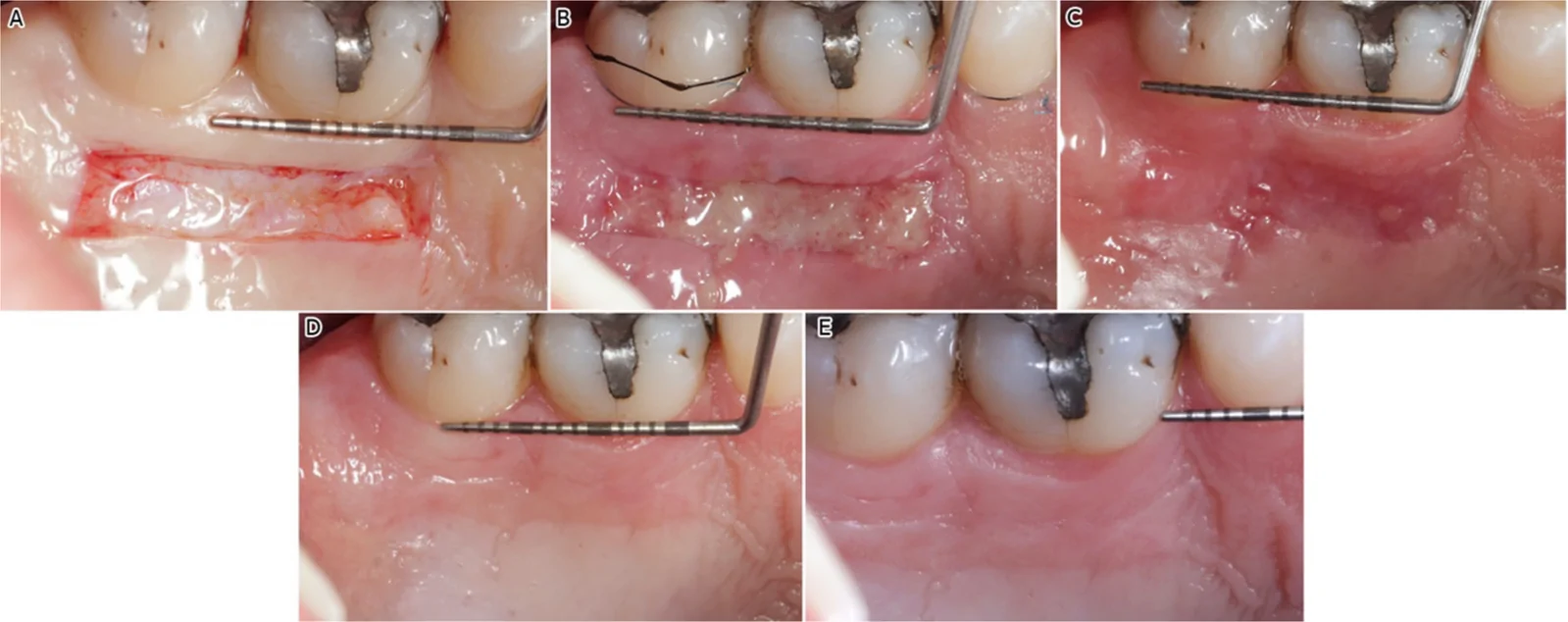
Palatal donor site in CG after gingival graft harvesting, illustrating the healing process over time. (**A**) Immediate postoperative aspect after graft harvesting (baseline). (**B**–**E**) Clinical appearance at 7, 14, 30, and 60 days postoperatively, respectively, with complete tissue healing observed at 60 days

**Fig. 7:**
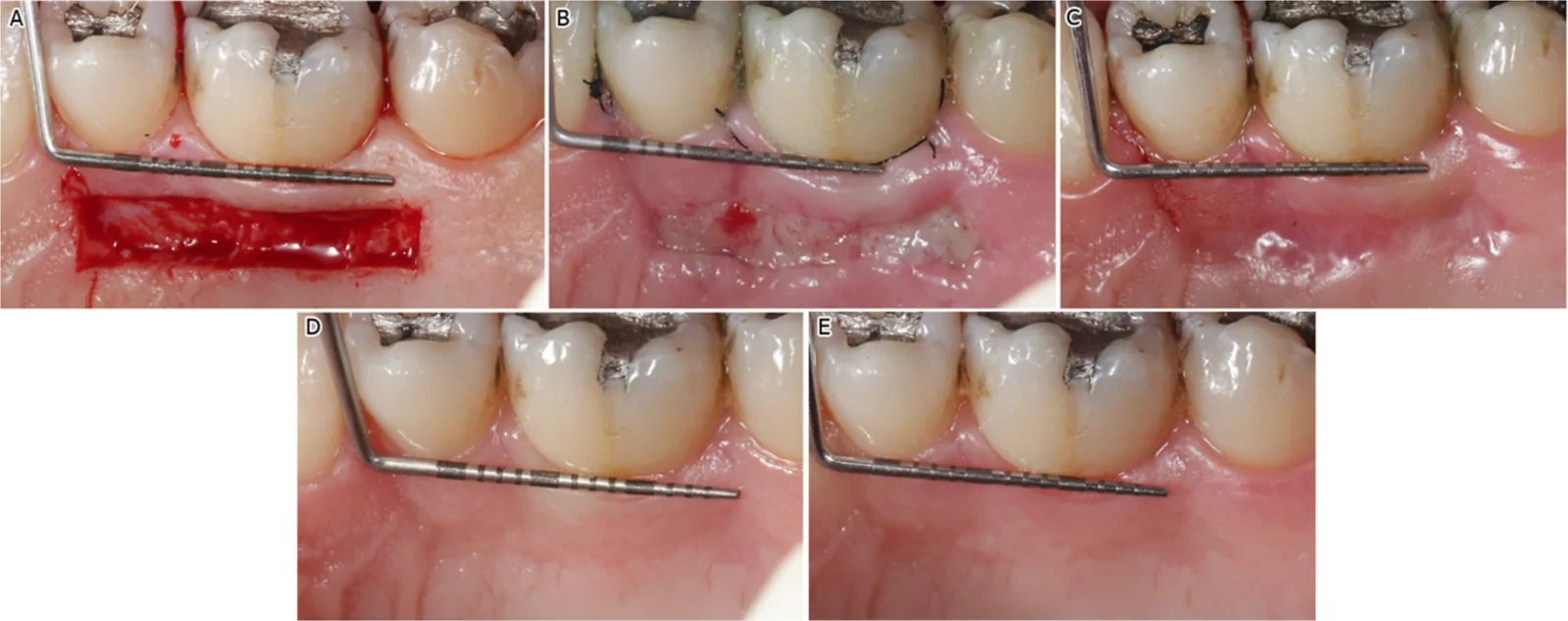
Palatal donor site in PHYG after gingival graft harvesting, illustrating the healing process over time. (**A**) Immediate postoperative aspect after graft harvesting (baseline). (**B**–**E**) Clinical appearance at 7, 14, 30, and 60 days postoperatively, respectively, with complete tissue healing observed at 60 days

At baseline, the wound area was 88.45 ± 13.69 mm² in the CG and 87.35 ± 13.53 mm² in the PHYG group. Both experimental groups demonstrated a progressive reduction in RWA over time, with statistically significant differences (*p* < 0.05) between 7 and 14 days postoperatively. Complete wound closure (RWA) was observed in both groups at 30 and 60 days. These findings are consistent with different uppercase letters assigned to time points. The PHYG showed numerically lower mean percentages of RWA compared with CG; however, no statistically significant intergroup differences were observed at any time point (*p* > 0.05). Lowercase letters indicate the absence of intergroup differences (Table 1) (Figs. 6 and 7).

No statistically significant intergroup differences were observed for TM at baseline and after 60 days (*p* > 0.05). TM decreased over time in both groups, from 3.67 ± 0.73 mm to 2.82 ± 0.55 mm in CG and from 4.04 ± 0.81 mm to 2.98 ± 0.71 mm in the PHYG (*p* < 0.05). Similarly, no significant differences were detected for WE between groups (*p* > 0.05). At 7 days, mean epithelialization (WE%) was 32.38 ± 12.28% in CG and 34.09 ± 11.08% in PHYG and after 14 days, 88.78 ± 8.08% and 91.97 ± 7.84%, respectively. A statistically significant (*p* < 0.05) increase in epithelialized area from 7 to 14 days was detected in both groups, as indicated by the different uppercase letters. Complete epithelialization (100%) was observed at 30 and 60 days. These results are consistent with the similar lowercase letter annotations between groups across all time points (Table 1), indicating comparable epithelialization patterns throughout the study period.

**Table 1:** Donor sites clinical parameters (RWA, TM, WE and RRA) at the different evaluation periods. Different uppercase letters indicate statistically significant differences over time (intragroup comparisons), whereas different lowercase letters indicate statistically significant differences between groups (intergroup comparisons) (*p* < 0.05)

Parameters	Groups	Baseline	7 days	14 days	30 days	60 days
RWA (%)	*CG*	100^A^	67.94 ± 12.28^B, a^	11.19 ± 7.84^C, a^	0^D^	0^D^
	*PHYG*	100^A^	65.72 ± 11.06^B, a^	8.00 ± 7.09^C, a^	0^D^	0^D^
TM (mm)	*CG*	3.67 ± 0.73^A, a^	-	-	-	2.82 ± 0.55^B, a^
	*PHYG*	4.04 ± 0.81^A, a^	-	-	-	2.98 ± 0.71^B, a^
WE (%)	*CG*	0^A^	32.38 ± 12.28^B, a^	88.78 ± 8.08^C, a^	100^D^	100^D^
	*PHYG*	0^A^	34.09 ± 11.08^B, a^	91.97 ± 7.84^C, a^	100^D^	100^D^
RRA (mm^2^)	*CG*	100^A^	79.47 ± 16.05^B, a^	63.03 ± 16.70^C, a^	27.66 ± 12.67^D, a^	0^E^
	*PHYG*	100^A^	71.71 ± 13.98 ^B, b^	54.12 ± 12.24^C, b^	21.91 ± 12.17^D, b^	0^E^

Data are presented as mean ± standard deviation (*SD*) or percentage values. *RWA* residual wound area, *TM* thickness of the masticatory mucosa and, *WE* degree of wound epithelialization, *RRA* residual remodeling area, CG control group, *PHYG* test group. Clinical outcomes were analyzed using two-way repeated measures ANOVA followed by Tukey’s post hoc test

RRA demonstrated statistically significant differences between groups and across time points (*p* < 0.05). PHYG consistently presented lower RRA values compared with CG, as indicated by distinct lowercase letters between groups at corresponding time points (7, 14 and 30 days) (Table 1). At 7 days, RRA values were 71.71 ± 13.98 mm² in the PHYG and 79.47 ± 16.05 mm² in CG. At 14 days, values were 54.12 ± 12.24 mm² (PHYG) and 63.03 ± 16.70 mm² (CG). At 30 days, RRA values were 21.91 ± 12.17 mm² in PHYG and 27.66 ± 12.67 mm² in CG. A statistically significant (*p* < 0.05) reduction in RRA from 7 to 30 days was observed in both groups, as indicated by the different uppercase letters. At 60 days, both groups presented RRA = 0 mm², indicating complete tissue resolution (Table 1). 

## Patient-centered outcomes

**Fig. 8:**
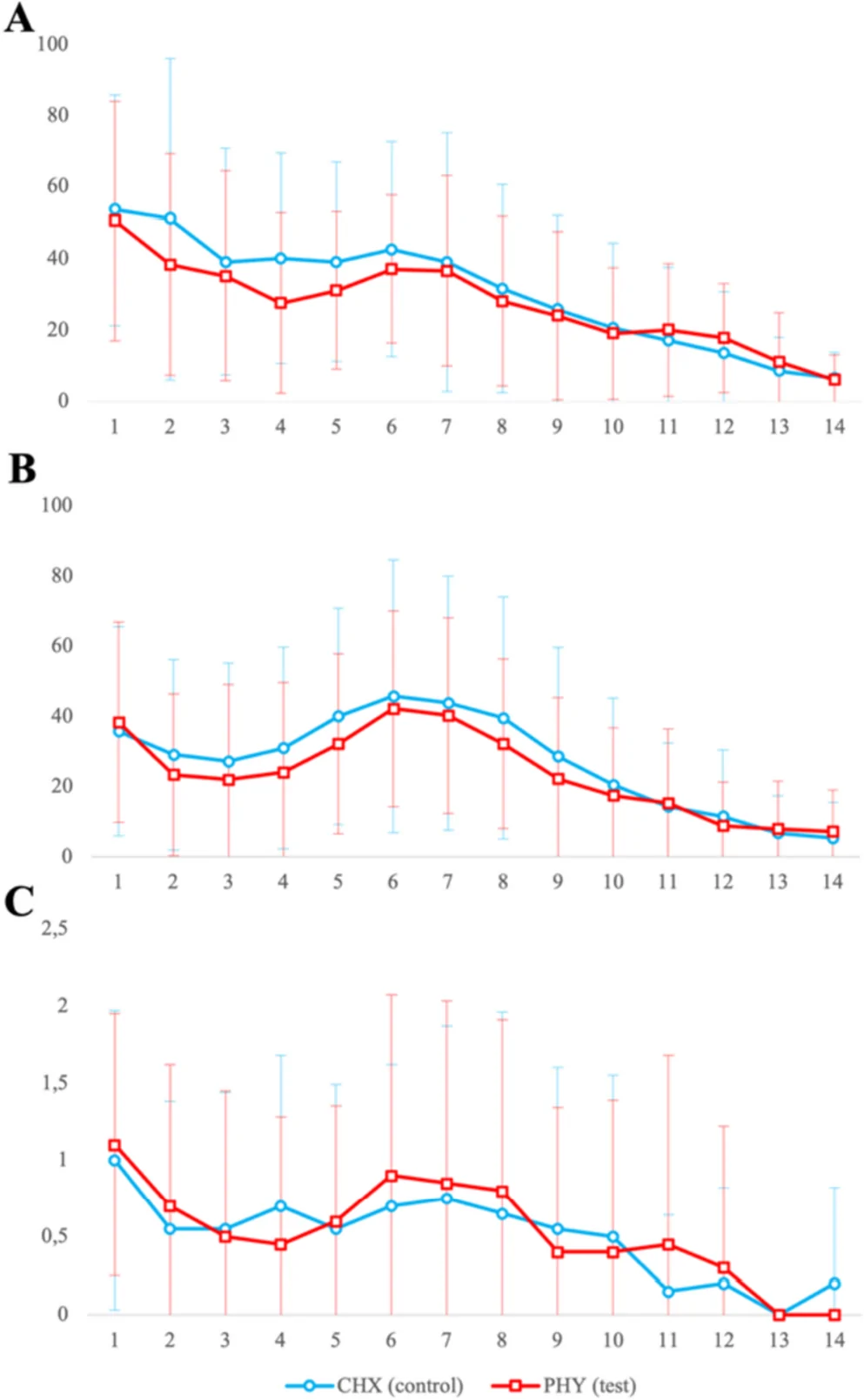
Mean (± standard deviation) patient-reported outcomes over 14 postoperative days for CG and PHYG. (**A**) Difficulty in chewing/eating (VAS); (**B**) Pain at the graft donor sites (VAS); (**C**) Analgesic consumption (number)

Patients adhered to the established protocol, completed the diaries appropriately, and did not present major complications such as hemorrhage, tissue necrosis, or adverse effects. Patient-centered outcomes included variables related to chewing difficulty, pain at palatal donor sites, and number of analgesics consumed per day (rescue medication). No statistically significant differences were detected between the CG and PHYG for any of the evaluated variables (Fig. 8). However, a significant effect of time was observed, indicating a positive postoperative evolution in patients’ subjective perceptions. Chewing ability improved progressively over the first 14 postoperative days. On day 1, mean scores were 54.29 ± 31.67 in CG and 51.43 ± 33.02 in PHYG. By day 14, these values decreased to 6.19 ± 7.23 and 5.71 ± 6.94, respectively. Pain reported at the donor sites followed a similar pattern demonstrated for chewing function. On postoperative day 1, mean pain scores were 35.71 ± 29.80 (CG) and 38.33 ± 28.56 (PHYG). On day 6, mean values were 45.71 ± 38.84 and 42.14 ± 27.91 for the CG and PHYG, respectively. From day 7 onward, a marked and continuous reduction in pain scores occurred, reaching mean values of 5.24 ± 10.18 (CG) and 7.14 ± 11.79 (PHYG) on day 14. The mean number of analgesics used was low from the early postoperative period for both groups, with a progressive reduction over time. Mean values of the number of analgesics consumed per day were 1.00 ± 0.95 (CG) and 1.10 ± 0.83 (PHYG) in day 1. In postoperative day 13, no rescue medication was required in either group.

OHIP-14 results showed no statistically significant differences between periods. The overall mean OHIP-14 score was 23.00 ± 6.84 at baseline and 23.05 ± 9.48 after 6 months of treatment (*p* > 0.05). This stability suggests that, overall, the performed procedures did not negatively affect patients’ oral health–related quality of life.

## Inflammatory biomarkers

Seven biomarkers in surgical wound exudates (IL-6, IL-10, PDGF-AA, PDGF-AB/BB, TGF-α, TNF-α, and VEGF-A) were quantified. Biomarker variation was calculated as the difference between concentrations at 7 and 3 days (Δ = day 7 − day 3). Positive values indicate an increase over time, whereas negative values indicate a decrease. Accordingly, the statistical analysis focused exclusively on intergroup comparisons of these variation values (Δ) (Tables 2 and 3).

**Table 2:** Comparisons between CG and PHYG (Δ = day 7 − day 3) for variables without normal distribution were performed using Wilcoxon test

Biomarkers	Group	Median	Q1 (25%)	Q3 (75%)	*p* value
IL-6	*CG*	-13.957	-36.569	107.708	0.517
	*PHYG*	-13.641	-39.158	55.631	0.517
IL-10	*CG*	-36.779	-57.345	0	0.013
	*PHYG*	17.485	-40.381	52.998	0.013
PDGF-AA	*CG*	-26.973	-52.242	2.830	0.268
	*PHYG*	-3.688	-43.410	94.197	0.268
PDGF-AB/BB	*CG*	-22.300	-88.842	246.407	0.404
	*PHYG*	-77.984	-89.234	340.422	0.404
TNF-α	*CG*	-18.217	-37.200	26.417	0.927
	*PHYG*	-15.086	-42.853	30.533	0.927
VEGF-A	*CG*	17.767	-19.730	112.769	0.706
	*PHYG*	10.572	-3.666	60.483	0,706

Data are presented as median and interquartile range (Q1–Q3). *CG* control group, *PHYG* test group, *IL-6* interleukin-6, *IL-10* interleukin-10, *PDGF-AA* platelet-derived growth factor-AA, *PDGF-AB/BB* platelet-derived growth factor-AB/BB, *TNF-α* tumor necrosis factor-alpha, *VEGF-A* vascular endothelial growth factor-A, *Q1* first quartile (25%), *Q3* third quartile (75%)

**Table 3:** Comparisons between CG and PHYG (Δ = day 7 − day 3) for variables with normal distribution were performed using the t test

Biomarker	Group	Mean ±	SD	*p* value
TGF α	*CG*	26.344	54.821	0.751
	*PHYG*	21.540	41.894	0.751

Data are presented as mean ± standard deviation (SD). *CG* control group; *PHYG*: test group, *TGF-α* = transforming growth factor-alpha, *SD* = standard deviation.

No intergroup differences were observed for most biomarkers (all *p* > 0.05), except for IL-10, which showed a significant difference between groups (*p* = 0.013). IL-10 levels were negative in CG (− 36.779) and positive in PHYG (17.485), as presented in Table 2.

For IL-6, PDGF-AA, PDGF-AB/BB, TNF-α, and VEGF-A, values differed numerically between groups; however, these differences were not significant (*p* = 0.517, *p* = 0.268, *p* = 0.404, *p* = 0.927, and *p* = 0.706, respectively). Similarly, no statistically significant difference was found for TGF-α between groups (*p* = 0.751). These results indicate that, except for IL-10, biomarker levels were comparable between groups. 

Despite variations in analyte levels, no statistically significant correlations were identified between changes in biomarker concentrations and clinical parameters (RWA, TM, WE and RRA), postoperative pain, or analgesic consumption across the evaluated postoperative time points.

## Quantitative Sensory Testing

The evolution of sensory thresholds was assessed in both experimental groups over time (baseline, 60 days, and 6 months) (Table 4). Analyses were performed separately for MDT and MPT. MDT and MPT data were log10-transformed to meet the assumptions required for parametric analysis. Statistical tests were conducted on the transformed data; however, for clinical interpretability, descriptive statistics were presented in the original scale as median (Q1–Q3).

**Table 4:** Mechanical detection threshold (MDT) and mechanical pain threshold (MPT) at palatal donor sites evaluated at baseline, 60 days, and 6 months. Different uppercase letters indicate statistically significant differences among time points based on post hoc comparisons for the main effect of time

Parameter (g/mm²)	Group	Baseline	60 days	6 months
MDT	CG	0.551 (0.300–2.65)^A^	0.565 (0.288–1.94)^A^	0.825 (0.275–1.23)^A^
MDT	PHYG	0.910 (0.470–2.47)^A^	0.717 (0.195–5.40)^A^	0.774 (0.322–1.62)^A^
MPT	CG	57.3 (19.7–110)^A^	34.4 (11.5–50.1)^B^	39.5 (26.8–58.9)^AB^
MPT	PHYG	48.5 (29.6–101)^A^	19.7 (17.9–50.1)^B^	27.6 (11.8–67.7)^AB^

Data are presented as median (Q1–Q3). *CG* control group, *PHYG* test group, *MDT* = mechanical detection threshold, *MPT* = mechanical pain threshold

MDT results showed no significant effects of time (*p* = 0.391), group (*p* = 0.473), or their interaction. The detection threshold at the donor site remained stable throughout the 6-month follow-up. Specifically, for MDT, all time points shared the same uppercase letter, indicating no significant differences over time.

In contrast, MPT demonstrated a statistically significant effect of time (*p* = 0.022), with a significant reduction at 60 days compared with baseline (*p* = 0.009), suggesting increased pain sensitivity in the early postoperative period. For MPT, baseline and 60-day evaluations presented different uppercase letters, reflecting the significant reduction observed at 60 days compared with baseline. However, this reduction was no longer significant at 6 months. No differences between groups were exhibited. The 6-month evaluation received the designation “AB”, indicating that it did not significantly differ from either baseline or 60 days. For MPT, a significant main effect of time was observed (*p* = 0.022), whereas no significant group effect (*p* = 0.934) or time × group interaction (*p* = 0.229) was found. Post hoc comparisons demonstrated a significant reduction at 60 days compared with baseline (*p* = 0.009), indicating transient postoperative hypersensitivity. However, this reduction was no longer significant at 6 months. Since no interaction effect was exhibited, this temporal pattern was similar in both groups.

The findings indicated that most sensory thresholds remained stable over time in donor areas of SCTG, except for MPT, which showed transient hypersensitivity at 60 days. None of the analyses revealed significant group effects or interactions, suggesting that the type of mouthwash or gel used did not significantly influence sensory recovery.

## Discussion

The present investigation revealed that PHYG and CG exhibited similar clinical outcomes during the healing process of palatal donor sites. PHYG demonstrated superior RRA outcomes, suggesting a more favorable tissue remodeling pattern during the healing process. However, this clinical difference did not influence patient-centered outcomes, since groups presented no statistically significant differences for chewing, pain and rescue medication. Clinical and patient outcomes demonstrated a significant effect of time, indicating a positive postoperative progression. IL-10 levels showed a significant increase in PHYG, suggesting a more favorable anti-inflammatory response compared with CG. Quantitative sensory testing revealed that most of the evaluated sensory thresholds remained stable over time but, donor sites exhibited transient hypersensitivity at 2 months.

Thus, both protocols promoted progressive healing of palatal donor sites, with the only significant difference being the lower RRA observed in the PHYG. An in vitro study showed that phthalocyanine group exhibited lower cytotoxicity and presented superior fibroblast migration compared to chlorhexidine [18]. These findings partially align with emerging evidence from wound-healing adjunctive therapies in palatal donor area, where the use of hyaluronic acid has demonstrated enhanced epithelialization and reduction in wound size compared with controls [42]. Considering topical agents with slow oxygen release for oral wound healing, a randomized clinical trial [43] demonstrated significantly improved healing indices and reduced postoperative pain in free gingival graft donor sites. These findings support the biological potential of therapies that enhance local oxygen availability, promoting accelerated re-epithelialization, possibly through the stimulation of angiogenesis and extracellular matrix production [43, 44]. Furthermore, photobiomodulation modalities have been associated with accelerated early wound healing and wound area reduction in donor sites, although with inconsistent effects on patient-reported pain outcomes [45, 46].

The absence of intergroup differences in patient-reported outcomes, despite the observed differences in RRA and IL-10 in PHYG, should be interpreted with caution. Clinical parameters such as RRA primarily reflect morphological aspects of tissue remodeling, whereas patient-centered outcomes, including pain and chewing discomfort, are influenced by complex neurobiological and psychosocial factors. In the present study, both groups exhibited relatively low levels of postoperative pain and analgesic consumption, which may have limited the ability to detect intergroup differences. Additionally, the split-mouth design may have contributed to a homogenization of subjective perceptions within the same individual. It is also important to consider that the biological effects of PHY, such as modulation of inflammatory mediators (e.g., increased IL-10), may be more closely associated with tissue remodeling phases rather than the acute inflammatory phase, which is more directly related to pain perception. These findings are in line with recent evidence [47, 48], which indicate that postoperative interventions for palatal donor sites frequently result in limited, inconsistent, or heterogeneous effects on patient-reported outcomes, despite showing improvements in clinical healing parameters. While PHYG was associated with improved morphological healing and modulation of inflammatory biomarker, these findings did not translate into measurable improvements in patient-centered outcomes. Therefore, the clinical significance of these observations remains to be established and should be confirmed in future studies.

Inflammatory biomarkers demonstrated a reduction in IL-6 levels in both groups, indicating a progressive resolution of the local inflammatory response, consistent with the transition from the inflammatory to the proliferative phase of wound healing [49, 50]. The increase in IL-10 in PHYG, suggested a more pronounced anti-inflammatory modulation, as this cytokine limits the production of pro-inflammatory mediators and contributes to tissue protection [51]. Despite this biochemical difference, no significant correlations were found between changes in biomarker levels and clinical parameters or patient-reported outcomes. This finding indicates that modulation of the local inflammatory response does not necessarily translate into measurable clinical differences. In this context, biomarkers such as IL-10 may reflect subtle immunomodulatory effects that are not directly captured by clinical parameters or patient-reported outcomes. The reductions in PDGF-AA, PDGF-AB/BB, and TNF-α are consistent with the resolution of early inflammation and progression of tissue repair, given the role of these mediators in cellular chemotaxis, fibroblast proliferation, and angiogenesis during the initial phases of healing [52]. Similarly, the increase in VEGF-A levels may be associated with angiogenic activity during the proliferative phase [53]. Therefore, the biomarker findings of the present study should be interpreted as indicative of underlying biological activity rather than direct predictors of clinical outcomes.

Another important parameter explored was quantitative somatosensorial evaluation. Studies investigating the sensory profile of gingival graft recipient and donor sites are scarce. However, qualitative analyses have already demonstrated sensory alterations that are compatible with the maintenance of oral function [25, 26]. The quantitative findings (MPT and MDT) of the present study are consistent with these outcomes [25, 26] that demonstrated a hypersensitive response in donor areas at 3 months, followed by a reduction in sensitivity at 6 months postoperatively. Similarly, the quantitative analysis showed that most of the evaluated sensory thresholds remained stable over time, although donor sites exhibited transient hypersensitivity (MPT) at 2 months. Sensory studies may yield controversial results, as they depend on individual pain tolerance thresholds and on the methods used for analysis. Nevertheless, the present findings indicated that although sensory alterations may occur in donor areas, they were transient and compatible with normal oral function. The observed temporary hypersensitivity reflects short-term sensory responses rather than persistent or clinically significant symptoms. This information is particularly important to ensure that patients are properly informed that experiencing minor sensory alterations during the postoperative period is normal and that these changes tend to resolve over time.

Despite the innovative and clinically relevant aspects of the present randomized clinical trial, some limitations could be observed. The large number of outcomes and statistical comparisons performed may increase the risk of type I error. In addition, the study was not specifically powered for secondary outcomes. A selected panel of immunological biomarkers was analyzed, which does not encompass the full spectrum of mediators involved in the wound-healing process. The use of nimesulide during the initial healing phase may have influenced the inflammatory biomarker analysis. Another limitation was the absence of evaluations of the interaction between the oral microbiome, biofilm dynamics, and host immunological response. Another potential limitation is that slight differences in gel color between formulations could have potentially influenced participant blinding, although efforts were made to minimize this risk through identical packaging and standardized handling procedures. Quantitative sensory assessment may carry an inherent degree of subjectivity due to interindividual variability in sensory perception. Nevertheless, the quantitative findings were consistent with the qualitative outcomes [25, 26], reinforcing the overall reliability of the results.

Despite these limitations, this study integrated clinical outcomes, patient-centered outcomes, quantitative sensory testing, and molecular biomarkers to comprehensively evaluate the effects of a topical oxygen-releasing therapy in oral wound healing. These outcomes present novel and clinically significant aspects to support biological strategies for optimizing postoperative care in periodontal plastic surgery.

## Conclusion

Within the limitations of this randomized clinical trial, both postoperative protocols (PHYG and CG) demonstrated comparable effectiveness in promoting palatal donor site healing following SCTG harvesting, with no clinically meaningful differences in clinical or patient-reported outcomes. The transient hypersensitivity observed during healing was self-limiting and did not translate into clinically relevant patient discomfort.

## Data Availability

The datasets generated and/or analyzed during the current study are not publicly available due to privacy and ethical restrictions but are available on reasonable request.
